# A Computational Fluid Dynamic Study on Efficiency of a Wavy Microchannel/Heat Sink Containing Various Nanoparticles

**DOI:** 10.3390/mi12101192

**Published:** 2021-09-30

**Authors:** Yacine Khetib, Hala M. Abo-Dief, Abdullah K. Alanazi, S. Mohammad Sajadi, Mohsen Sharifpur, Josua P. Meyer

**Affiliations:** 1 Mechanical Engineering Department, Faculty of Engineering, King Abdulaziz University, Jeddah 80204, Saudi Arabia; 2 Center Excellence of Renewable Energy and Power, King Abdulaziz University, Jeddah 80204, Saudi Arabia; 3 Department of Chemistry, College of Science, Taif University, P.O. Box 11099, Taif 21944, Saudi Arabia; h.abodeif@tu.edu.sa (H.M.A.-D.); aalanaz4@tu.edu.sa (A.K.A.); 4 Department of Nutrition, Cihan University-Erbil, Kurdistan Region, Iraq; smohammad.sajadi@gmail.com; 5 Department of Phytochemistry, SRC, Soran University, KRG, Iraq; 6 Department of Mechanical and Aeronautical Engineering, University of Pretoria, Pretoria 0002, South Africa; josua.meyer@up.ac.za; 7 Department of Medical Research, China Medical University Hospital, China Medical University, Taichung 404, Taiwan

**Keywords:** wavy microchannel heat sink, two-phase model, nanofluid, numerical study

## Abstract

In this paper, a common and widely used micro-heat sink (H/S) was redesigned and simulated using computational fluid dynamics methods. This H/S has a large number of microchannels in which the walls are wavy (wavy microchannel heat sink: WMCHS). To improve cooling, two ($Al_{2}O_{3}$ and CuO) water-based nanofluids (NFs) were used as cooling fluids, and their performance was compared. For this purpose, studies were carried out at three Reynolds numbers (Re) of 500, 1000, and 1500 when the volume percent (*φ*) of the nanoparticles (NPs) was increased to 2%. The mixture two-phase (T-P) model was utilized to simulate the NFs. Results showed that using the designed WMCHS compared to the common H/S reduces the average and maximum temperatures (T-Max) up to 2 °C. Moreover, using the $Al_{2}O_{3}$ NF is more suitable in terms of WMCHS temperature uniformity as well as its thermal resistance compared to the CuO NF. Increasing the *φ* is desirable in terms of temperature, but it enhances the pumping power (PP). Besides, the Figure of Merit (FOM) was investigated, and it was found that the value is greater at a higher volume percentage.

## 1. Introduction

Nowadays, many available devices are intelligent and contain a variety of electronic components. One of the substantial obstacles in these electronic devices is concerned with the overheating issue during operation. These devices get hot by consuming electricity and performing their functions at the same time. This situation results in the consumption of electricity. In these components, overheating causes damage to the devices or burns these components. Therefore, it is essential to find a way to cool these devices. Using H/S is one of the best solutions for cooling different electronic components [1,2]. Heat sinks are established in a wide range of shapes and applications. Many of these heat sinks are cooled by airflow, and they have various applications [3,4]. However, in some devices with very high heating, the airflow is not sufficient for the cooling process. Water and fluids are utilized for the cooling process. Therefore, researchers have used different fluids in their research according to the conditions of their problem. [5,6,7]. According to the results of various research, the application of nanofluid (NF) can improve the thermal efficiency in heat exchangers [8,9,10,11]. Nanotechnology has been considered by researchers for many years [12,13,14,15,16]. The thermal conductivity in NF is higher than in fluids such as water [17,18,19,20]. Several researchers have employed NFs in their investigations [21,22,23,24]. Heat sinks are not an exception, and NFs are commonly used in heat sinks [25,26,27,28,29]. Bahiraie and Heshmatian [30] employed commercial software to numerically simulate the flow of a specific NF in a H/S. They changed the percentage of nanoparticles (NPs) and the inlet flow rate. The results revealed that growing the Reynolds number (Re) and volume percentage of the NPs decreases the maximum H/S temperature. By increasing the one percent by volume of water-based Na, the maximum temperature (T-Max) has dropped by 2.21 °C.

Ambreen and Kim [31] developed a numerical solution, and they investigated the thermal efficiency of a rectangular micro-H/S using titanium oxide NFs in water. In this study, several pin fins with different shapes were placed on the H/S. They found that circular pin fins, especially in higher Re numbers, could cool the H/S more than other shapes of pin fins. Additionally, an increase in the Re leads to the enhancement of the heat transfer from the H/S to the fluid. Alfaryjat et al. [32] employed a numerical method to investigate a micro-H/S containing various microchannels. They utilized different types of NPs in their study, including SiO_2_, CuO, and Al_2_O_3_. In this study, water fluid was used as the base fluid. They evaluated the effects of these three types of NFs on a micro-H/S. This procedure was performed by changing the Re in the slow flow range. These three types of NPs had different diameters. The results demonstrated that the temperature gradient formed in the H/S is affected by this diameter. Wu et al. [33] conducted a numerical study on a rectangular H/S considering many microchannels. The thermal efficiency of the H/S was analyzed by flowing various NFs in the H/S. Similar to many other heat sinks mentioned above, constant heat flux was applied from the floor. The analysis results demonstrated that the use of a hybrid NF of copper and alumina in water dramatically dropped the temperature of the H/S in comparison with applying other NFs. The results of studies on the channels and pipes revealed that the use of wavy walls instead of a flat wall improves heat transfer [33,34,35,36,37,38]. In one of these articles, Vanaki et al. [38] studied the impact of wavy walls on a macro-sized channel. They utilized silicon oxide NPs in the study. They found that an intensification in the NF flow rate leads to enhancement of the heat transfer. However, this process simultaneously intensifies the pressure drop, which is an undesirable phenomenon.

According to the review of previous articles, the temperature of electronic components such as CPU increases as a result of their operation, which damages them. Hence, due to their micro and millimeter dimensions, cooling them is very complicated. Thus, in this paper, the geometry of a MHS was investigated by changing its microchannel walls. Two widely used NFs of $Al_{2}O_{3}$ and CuO/waters were utilized as the operating fluid. The effect of using these two NFs was examined. The two-phase (T-P) method was employed to better simulate the NFs; the effect of NFs diameter was also studied. The output and loss exergies, as well as the second law efficiency, are the other issues studied. In the end, the thermal efficiency of the MHS was investigated by increasing the volume percent (φ) of each NP up to 2% in the Re range of 500 to 1500. The innovation of the present article is the use of wavy channel walls in heat sink using alumina/water and copper oxide/water nanofluids.

## 2. Problem Definition

**Figure 1:**
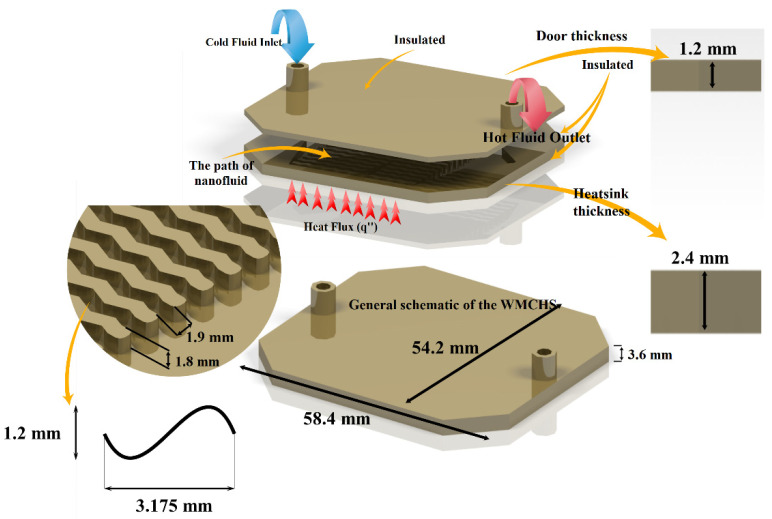
The model and boundary conditions applying to the geometry of the problem.

As displayed in Figure 1, the micro-H/S has a circular output and input placed on its top. The material of this H/S is aluminum, and it includes the principal part and upper part. The principal part of the H/S consists of 20 microchannels. The walls of these microchannels are wavy. The H/S has heights of 0.24 and 0.36 mm without and with considering the door, respectively. The inlet and outlet of the fluid have the same diameter of 0.19 mm. In the H/S, two different states are considered: (I) flowing copper/water oxide NF and (II) flowing water/alumina. The range of volume percentage of NPs was from 0 to 2%. These values were evaluated by the Re of 500, 1000, and 1500. A region under the H/S receives a constant flux from the operation of a Core i7 CPU.

## 3. Governing Equations

### 3.1. T-P Mixture Equations

The equations inside the WMCHS are in the form of a T-P mixture model, which are presented here. These equations have been written for the permanent flow of a Newtonian and incompressible fluid. The equations of conservation of mass, momentum, and energy in the T-P mixture model are solved for the mixture as the same as the volume percent equation for the second phase [39].

(1) $$\nabla \cdot \left(\rho_{mi}{\vec{v}}_{mi}\right)=0$$

(2) $$\nabla \cdot \left(\rho_{mi}{\vec{v}}_{mi}\cdot \nabla {\vec{v}}_{mi}\right)=-\nabla P_{mi}+\nabla \cdot \left(\mu_{mi}\nabla {\vec{v}}_{mi}\right)+\nabla \cdot \left(\overset{n}{\underset{k=1}{\sum }}\varphi_{k}\rho_{k}{\vec{v}}_{dr,k}{\vec{v}}_{dr,k}\right).\;$$

(3) $$\nabla \cdot \left(\overset{n}{\underset{k=1}{\sum }}\varphi_{k}\rho_{k}c_{p,k}{\vec{v}}_{k}T\right)=\nabla \cdot \left(k_{mi}\nabla T\right)\;$$

where $\vec{v}$, *T* and *P* referred to velocity, temperature, and pressure, ρ and *k* are density and thermal conductivity, and μ and $c_{p}$ are viscosity and heat capacity. The subscript *mi* indicates mixture. The amount of the average mixture velocity, density, viscosity, and thermal conductivity can be obtained by the following equations [40]:

(4) $${\vec{v}}_{mi}=\frac{\sum_{k=1}^{n}\varphi_{k}\rho_{k}{\vec{v}}_{k}}{\rho_{mi}}$$

(5) $$\rho_{mi}=\overset{n}{\underset{k=1}{\sum }}\varphi_{k}\rho_{k}$$

(6) $$\begin{array}{c} \mu_{mi}=-0.4892+\frac{26.9036}{T}+0.6837\varphi +\frac{24.1141}{T^{2}}-0.1785\varphi^{2}+0.1818\frac{\varphi }{T}+27.015\frac{\varphi^{2}}{T^{2}}+0.0132\varphi^{3}-2940.1775\frac{\varphi }{T^{3}}\;\;\;\;\;\;\;\;\left(Al_{2}O_{3}\right)\\ \mu_{mi}=-0.4262+\frac{8.4312}{T}+0.898\varphi +\frac{524.7147}{T^{2}}-0.2217\varphi^{2}-4.7329\frac{\varphi }{T}+70.3105\frac{\varphi^{2}}{T^{2}}+0.0176\varphi^{3}-5559.4641\frac{\varphi }{T^{3}}\;\;\;\;\;\;\;\;\left(CuO\right)\\ \frac{k_{mi}}{k_{f}}=1.0+1.0112\varphi +2.4375\varphi \left(\frac{47}{d_{p}\left(nm\right)}\right)-0.0248\varphi \left(\frac{k_{p}}{0.613}\right)\;\;\;\;\;\;\;\;\left(Al_{2}O_{3},\;CuO\right) \end{array}$$

The equation of volume percentage is formulated as follows:

(7) $$\nabla \cdot \left(\varphi_{p}\rho_{p}{\vec{v}}_{mi}\right)=-\nabla \cdot \left(\varphi_{p}\rho_{p}{\vec{v}}_{dr,p}\right)$$

Additionally, drift viscosity is represented for NP as follows (it is for the *k*-th phase):

(8) $${\vec{v}}_{dr,k}={\vec{v}}_{pf}-\overset{n}{\underset{i=1}{\sum }}\frac{\varphi_{k}\rho_{k}}{\rho_{mi}}{\vec{v}}_{fk}$$

The sliding velocity is defined as the second phase velocity that is dependent on the first phase.

(9) $${\vec{v}}_{pf}={\vec{v}}_{p}-{\vec{v}}_{f}$$

(10) $${\vec{v}}_{pf}=\frac{\rho_{p}d_{p}^{2}\left(\rho_{p}-\rho_{mi}\right)}{18\mu_{f}f_{\mathrm{drag}}\rho_{p}}a$$

(11) $$f_{\mathrm{drag}}=[\begin{array}{c} 1+0.15Re_{p}^{0.687},\;Re_{p}\leq 1000\\ \;\;0.0183Re_{p}^{0.687},\;Re_{p}>1000 \end{array}$$

In the above equations, the *p* and *f* indices are related to the NP and base fluid. Additionally, the gravitational acceleration is defined as follows:

(12) $$a=g-\left({\vec{v}}_{mi}\cdot \nabla \right){\vec{v}}_{mi}$$

The value of g is not considered if the gravitational acceleration is ignored.

The standard k-ε turbulence model is used to simulate turbulent flow. This model is suitable for turbulent flow with low velocity. In the following, the governing equations of the standard k-ε turbulence model are presented:

(13) $$\frac{\partial \left(u\rho k\right)}{\partial x}=\frac{\partial }{\partial x}\left[\left(\mu +\frac{\mu_{t}}{\sigma_{k}}\right)\frac{\partial k}{\partial x}\right]+\mu_{t}\left(\frac{\partial v}{\partial x}+\frac{\partial u}{\partial y}\right)\frac{\partial v}{\partial x}-\rho \varepsilon$$

(14) $$\frac{\partial \left(u\rho \epsilon \right)}{\partial x}=\frac{\partial }{\partial x}\left[\left(\mu +\frac{\mu_{t}}{\sigma_{\varepsilon }}\right)\frac{\partial \varepsilon }{\partial x}\right]+C_{1}\frac{\varepsilon }{k_{f}}\mu_{t}\left(\frac{\partial v}{\partial x}+\frac{\partial u}{\partial y}\right)\frac{\partial v}{\partial x}-\rho C_{2}\frac{\varepsilon^{2}}{k_{f}}$$

where $\mu_{t}$ is the dynamic viscosity for turbulent flow (kg/m·s). k and ε turbulence kinetic energy and turbulent dissipation rate, respectively. $C_{1}$ and $C_{2}$ were constant values that are equal to 1.44 and 1.92, respectively. $\sigma_{\varepsilon }$ and $\sigma_{k}$ are the turbulent flow Prandtl number and are 1.3 and 1.0, respectively.

Table 1 illustrates the properties of pure fluid and alumina and copper NPs. In this case, *d* is the diameter of the NPs (its unit is nm).

### 3.2. Boundary Condition

The existing boundary conditions for the H/S are displayed in Figure 1. The inlet and outlet of the fluid have the same diameter of 0.19 mm. In this situation, the fluid enters the H/S with a constant velocity at the temperature of 300° Kelvin, and then it exits from the H/S at the atmospheric pressure. A constant heat flux of 88,000 W/m^2^ is applied to the aluminum H/S in the surface area of 14.71 cm^2^ (Figure 1) These thermal boundary conditions operate as the cooling system for an Intel Core i7 CPU and its similar components. It is assumed that all the outer walls of the H/S are insulated, except the region subjected to the constant heat flux.

## 4. Numerical Process, Mesh Independency and Validation

After designing the geometry and applying mesh, the control volume and SIMPLE algorithm solved the velocity-pressure coupling. After activating the T-P mixture model, the first-order upwind scheme was employed to solve the momentum and energy equations. The convergence criterion was 10^−7^ for the equations. To use the models for thermal conductivity and viscosity, an in-house UDF code has been added to the software to calculate the values of thermal conductivity and viscosity correlations more accurately.

**Figure 2:**
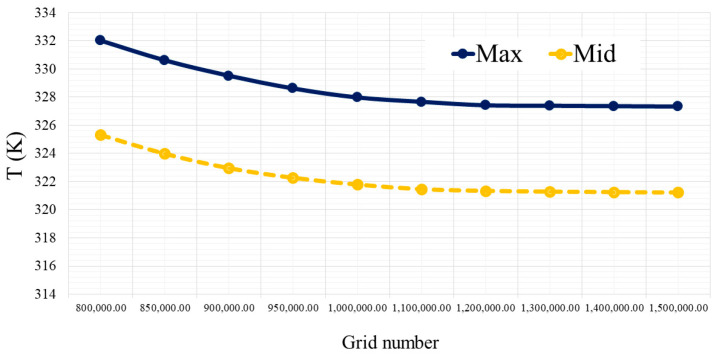
Grid independency study comparing the output temperature of the WMCHS based on the number of grids.

By examining various grids on the geometry, the grid with 1,220,000 elements was chosen as the grid. Figure 2 shows the comparisons between different factors (e.g., average temperature and T-Max of H/S) for selecting mesh according to the number of elements considering two Re numbers and 2% percent by volume of NPs. According to this figure, the mentioned mesh was chosen for this geometry.

**Figure 3:**
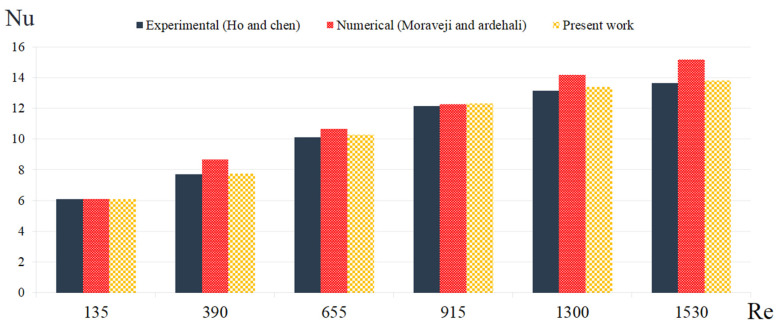
Results of current study in comparison of the literature [43] (experimental) and [39] (numerical).

To validate and investigate the accuracy of the performed numerical work results, a comparison between the current research work and a number of similar articles was carried out. For this goal, the findings were compared to a T-P numerical method by Moraveji and Ardehali [39] and an experimental work by and Ho and Chen [43]. As displayed in Figure 3, the difference between the findings of the T-P method used in this work and ref [43] is small.

## 5. Thermal Analysis

In the following, the parameters, which are utilized to scrutinize the thermal efficiency of the WMCHS, are introduced. One of the most important of these parameters is the heat transfer coefficient expressed as follows [29]:

(15) $$h=\frac{q\prime \prime }{T_{\mathrm{Mid}}-T_{m}}$$

where T_m_ is $\frac{T_{in}-T_{out}}{2}$ ($T_{in}$: input temperature of the fluid, $T_{out}$: output temperature of the fluid). $T_{\mathrm{Mid}}$ is the average temperature of the part of the WMCHS constant flux and q″ is the heat flux applying on the WMCHS, which is equal to 88,000 $W/m^{2}$.

The pumping power (PP) indicating the energy required to move the fluid in the WMCHS is introduced in the below relations.

(16) $$\mathrm{PP}=\;\dot{Q}\Delta P$$

where $\;\dot{Q}$ denotes the volume flow rate of the fluid and ΔP is the pressure difference at the input and output of the WMCHS. The other parameter investigated is the Figure of Merit (FOM). This relation is applied when the NF is used instead of a normal fluid; also, it is presented as follows.

(17) FOM=hnfhfΔPnfΔPf

The other two important parameters which are useful to investigate the thermal efficiency of the WMCHS are the thermal resistance value and its temperature uniformity. The first parameter is an indication of thermal resistance of WMCHS against heat transfer, and the second one is the temperature distribution of the WMCHS. The relations concerning the aforementioned parameters are as follows, respectively. In fact, according to several investigations, the lower the value of these two parameters, the lower the thermal resistance of the WMCHS as well as the higher the temperature uniformity.

(18) $$R=\frac{T_{Mid}-T_{in}}{q\prime \prime }$$

(19) $$\theta =\frac{T_{Max}-T_{min}}{q\prime \prime }\;$$

In the above relations, the subscripts Max and Min indicate the T-Max and minimum temperature on the surface of the WMCHS constant flux.

The Re, which is written as follows, is utilized to show the fluid velocity.

(20) Re=ρfvlμf

## 6. Results and Discussion

In the simulation process, the NFs water/$Al_{2}O_{3}$ and water/CuO oxide were compared to the pure water at the Res of 500, 1000, and 1500. Furthermore, the φ of NPs was increased to 2% and the results were expressed as follows.

**Figure 4:**
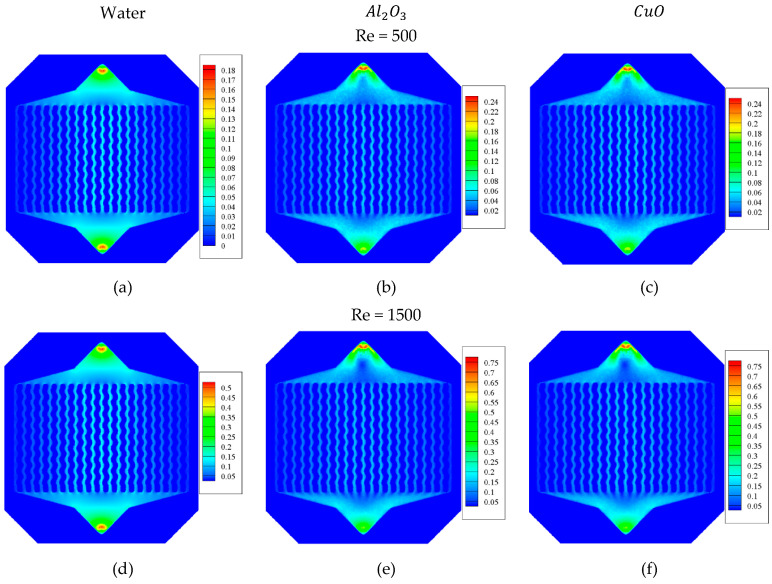
The velocity field for the $Al_{2}O_{3}$ and CuO water-based NFs at the Res of 500 (**a**–**c**) and 1500 (**d**–**f**) on the middle plane of the WMCHS.

The velocity field for the $Al_{2}O_{3}$ and CuO water-based NFs at the Res of 500 and 1500 on the middle plane of the WMCHS is presented in Figure 4. As can be seen, the maximum velocity in the WMCHS increased. This maximum velocity occurs at the input and output parts of the fluid, where the crossing section of the fluid is smaller than the other parts. The fluid’s velocity becomes lower when it passes through the input region and reaches the wider WMCHS area. Moreover, the fluid flows faster in the middle microchannels compared to the side ones. In fact, in comparison with the side microchannels, more fluid passes through the middle microchannels of the WMCHS.

**Figure 5:**
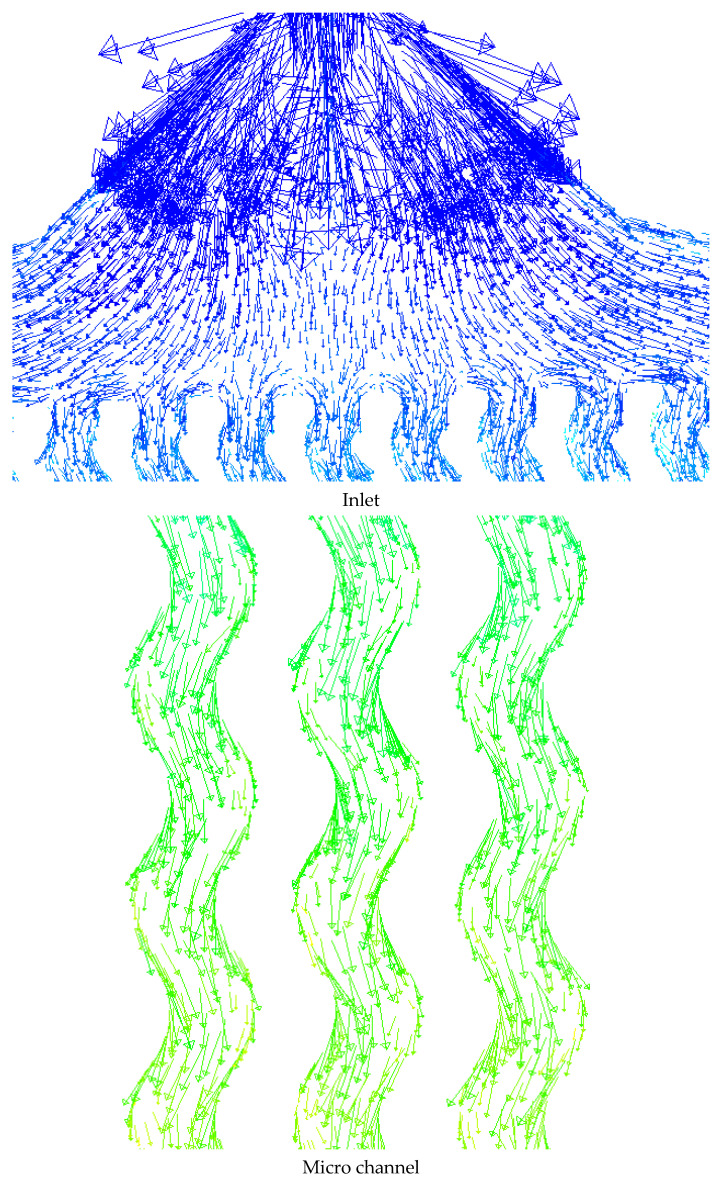
Velocity vectors at the fluid inlet and between the microchannels.

Velocity vectors at the fluid inlet and between the microchannels are shown in Figure 5. It can be seen that there is a high velocity change at the inlet due to the sudden enhancement in the flow rate. Initially, at the inlet, the fluid velocity is high due to the narrow passage of the fluid, leading to long vectors. With the change of flow direction in the heat sink and the enhancement of the flow area, the size of the vectors becomes smaller, indicating a decrease in velocity. The fluid motion between the microchannels and its collision with the walls show that the fluid has more collision with smooth walls in the corrugated walls.

**Figure 6:**
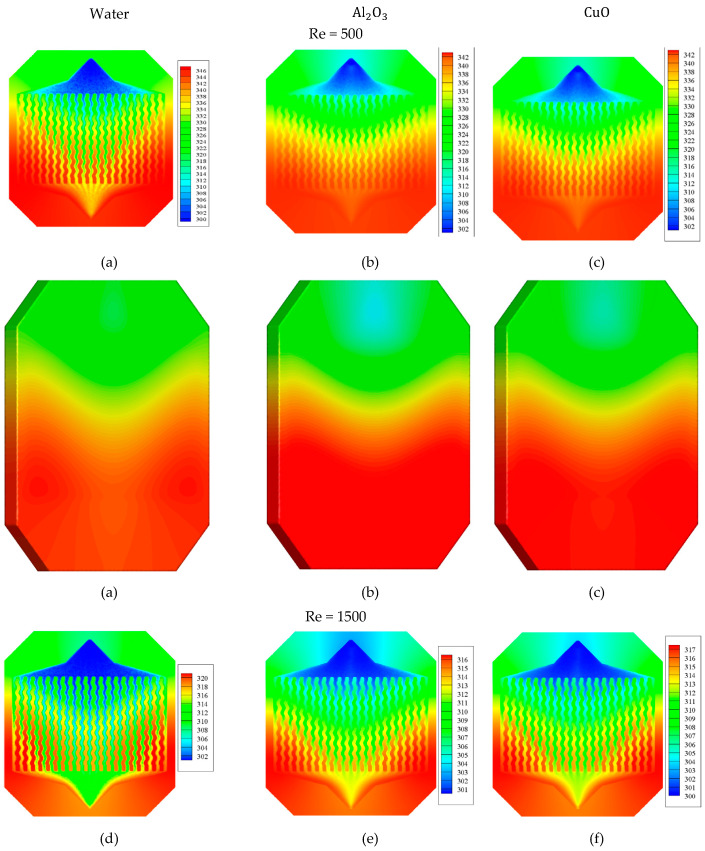

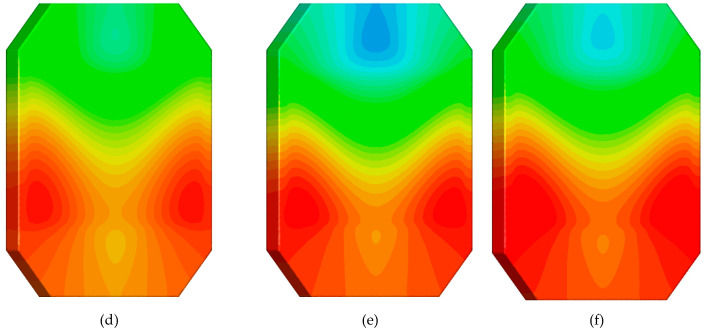
Three-dimensional temperature contour at the middle plane (upper) and the part underneath the WMCHS (bottom) for $Al_{2}O_{3}$ and CuO water-based NFs at Re = 500 (**a**–**c**) and 1500 (**d**–**f**).

Figure 6 shows the temperature contour at the middle plane (upper) and the part underneath the WMCHS (bottom) for the pure water and the water/$Al_{2}O_{3}$ and water/CuO NFs at the Re numbers of 500 and 1500. As observed, augmenting the Re decreases the T-Max and overall temperature of the WMCHS. In addition, increasing the velocity of the fluid leads to improving heat transfer and better cooling the WMCHS, which are observable in all the fluids. Compared to pure water, using NFs better improves the cooling process of the WMCHS. It should also be mentioned that between two NFs used in the current research, the water/$Al_{2}O_{3}$ NF has better performance in the cooling process. Actually, the presence of the NPs in the fluid flow leads to heightening its thermal conduction resulting in improvement of heat transfer, and finally, better cooling in the WMCHS. WMCHS has a lower temperature in its middle part where the fluid passes faster, while it has a higher temperature in its side parts where the fluid passes more slowly.

**Figure 7:**
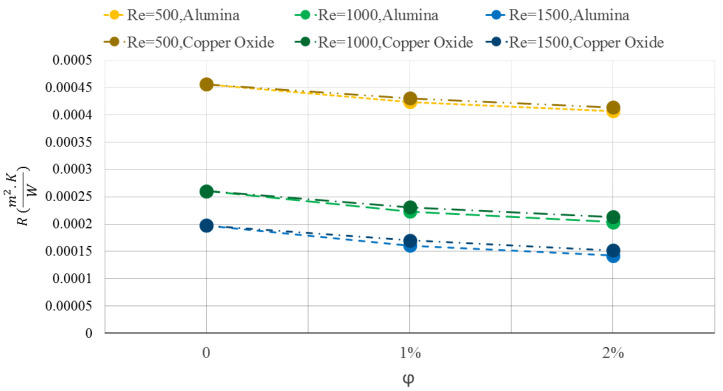
WMCHS thermal resistance for Water/$Al_{2}O_{3}$ and Water/CuO NFs at three different Res of 500, 1000, and 1500.

Figure 7 indicates the thermal resistance of the WMCHS for water/$Al_{2}O_{3}$ and water/CuO NFs at three different Res of 500, 1000, and 1500. As can be seen, the addition of both NPs decreases the value of the WMCHS thermal resistance, which is desirable. Compared to the water/CuO Na, using the $Al_{2}O_{3}$ NPs in the base fluid creates lower thermal resistance in the WMCHS. Increasing the Re also decreases the thermal resistance of the WMCHS. The average temperature decrease in the WMCHS leads to a decline in its thermal resistance by enhancing the Re and adding NPs.

**Figure 8:**
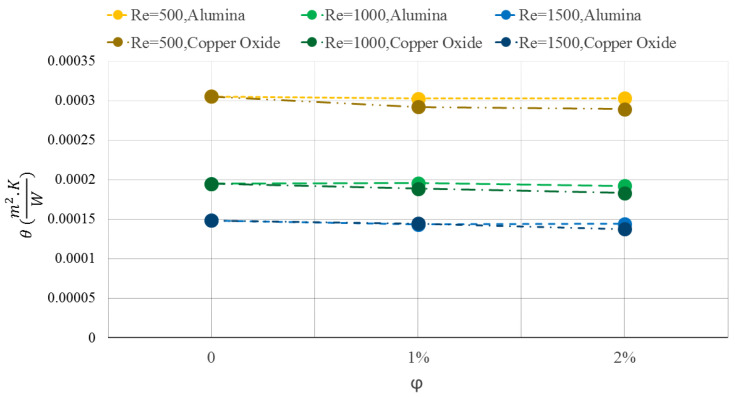
Temperature uniformity values on WMCHS for two different NFs at three Res.

Figure 8 shows the temperature uniformity of the WMCHS for two different NFs at three Res. The addition of the NPs to the base fluid leads to improving the temperature uniformity as well as closing the T-Max and minimum temperature of the WMCHS to each other. Moreover, according to the graph illustrated in Figure 8, the water/$Al_{2}O_{3}$ NF has better operation than the water/CuO one. Therefore, it creates better uniformity. The lower value of θ means closing the T-Max and minimum temperature of the WMCHS to each other, which is desirable in the WMCHS design. Increasing the Re decreases the θ values, which is the reason for the severe decrease in the T-Max.

**Figure 9:**
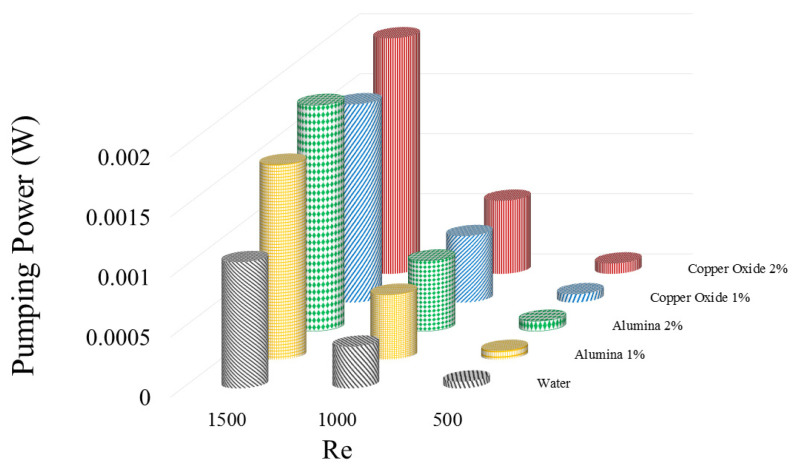
PP values required for moving the fluid having different Res for water fluid and water/$Al_{2}O_{3}$ and water/CuO NFs at two various volume percentages.

Figure 9 illustrates the PP values required for moving the fluid having different Re numbers for the water fluid and water/$Al_{2}O_{3}$ and water/CuO NFs at two various volume percentages. The PP greatly enhances by increasing the Re owing to the simultaneous increase in the flow rate and pressure drop in the WMCHS. Moreover, the addition of the NPs increases the PP due to heightening the pressure drop. As can be seen, there is a drastic pressure drop at the higher Res by adding the NPs to the base fluid. On the other hand, adding the NPs and increasing them heightens the pressure drop. Besides, increasing the NPs dimensions requires a higher PP than the smaller NPs. It is then observed that the highest required PP is related to the 2 vol% water/CuO NF at Re=1500.

Table 2 shows the pressure drop at different Reynolds numbers for two different nanoparticles and different volume percentages of nanofluids. It can be seen that the addition of nanoparticles enhances the amount of pressure drop in the heat sink. An increment in the Reynolds number also increases the amount of pressure drop in the heat sink. An enhancement in the Reynolds number means an increment in the velocity gradient in the heat sink, which ultimately leads to an increase in the pressure drop. Additionally, due to the higher density of copper oxide than alumina nanoparticles, the amount of velocity difference or lift velocity created for nanoparticles in the two-phase mixture method is different. Copper oxide nanoparticles have a higher density, and as a result increase the density of the nanofluid, which can lead to an enhancement in the system pressure drop.

**Figure 10:**
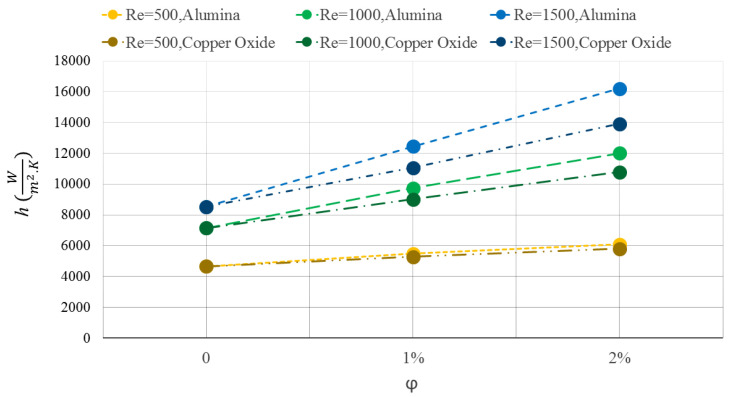
Convection heat transfer coefficient between the WMCHS and the fluid for water/$Al_{2}O_{3}$ and water/CuO NFs at three different Res.

Figure 10 indicates the convection heat transfer coefficient between the WMCHS and the fluid for the water/$Al_{2}O_{3}$ and water/CuO NFs at three different Res. Using the NFs, especially with higher volume percentages, greatly increases the heat transfer coefficient. Increasing the fluid thermal conductivity, particularly for the $Al_{2}O_{3}$ NPs, highly improves heat transfer at the contact surface of the wall and the fluid. The improvement of the heat transfer is increased by adding the NPs at the higher Res. Increasing the Re heightens the heat transfer coefficient due to speeding up the fluid on the surface of the microchannel walls.

**Figure 11:**
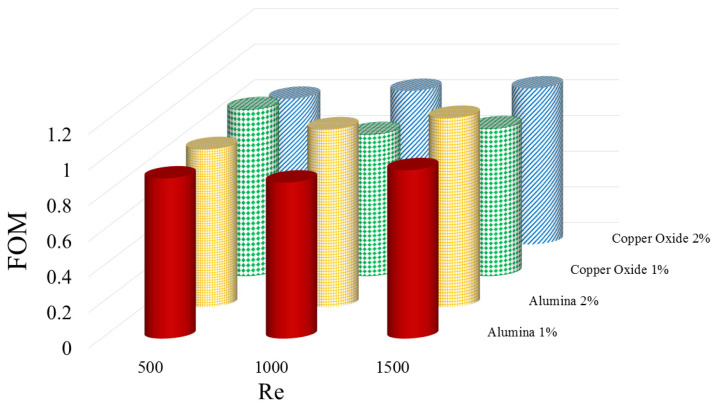
FOM for 1 vol and 2 vol% $Al_{2}O_{3}$ and CuO NFs at Re=500, 1000, and 1500.

Figure 11 shows FOM for 1% and 2 vol% $Al_{2}O_{3}$ and CuO NFs at three various Res of 500, 1000, and 1500. As can be generally seen using smaller dimensions, NPs having a higher thermal conduction coefficient creates a higher FOM value than the other NPs. This is as a result of greater enhancement of the heat transfer coefficient using this NP and its lower pressure drop, which leads to improving the FOM of the $Al_{2}O_{3}$ NP compared to the CuO one. Hence, the maximum FOM is related to the $Al_{2}O_{3}$ NF at 2 vol%. Adding more NPs improves the FOM, which shows using higher volume percentages of NPs is more desirable.

## 7. Conclusions

In this paper, the cooling of a new micro-heat sink (H/S) designed using two different types of nanofluids (NFs) was investigated by employing the T-P mixture method. The NFs were water/$Al_{2}O_{3}$ and water/CuO with 2 vol% nanoparticles (NPs) in the base fluid. Moreover, three different Re numbers of 500, 1000, and 1500 were chosen. The designed H/S had a number of wavy walls microchannels (WMCHS). The results showed that using the wavy walls in the microchannels rather than the simple ones decreases the T-Max and average temperatures of the WMCHS up to 2 °C in a similar condition compared to the previous research works [30,44]. As a result, WMCHSs presented better thermal resistance and uniformity in comparison with the similar H/Ss. However, the wavy walls would add to pumping power compared to straight walls. The results of using two different dimensions NPs at various Re numbers are as follows:

1. Using both the investigated NFs instead of the pure water decreases the thermal resistance of the WMCHS and creates more temperature uniformity in it. Moreover, using the $Al_{2}O_{3}$ NP is more suitable in the cooling operation of the H/S;
2. The addition of the NPs, especially $Al_{2}O_{3}$, increases the heat transfer coefficient. Furthermore, the convection heat transfer coefficient increase is more noticeable at the higher Res;
3. The addition of the higher diameter NPs, especially CuO, leads to increasing the PP required for moving the fluid. Compared to other NPs, using these NPs has also increased the power consumption cost significantly;
4. Increasing the φ increases the Figure of Merit value so that its maximum value occurs at a 2 vol% $Al_{2}O_{3}$ NF;
5. Increasing the Re decreases the thermal resistance of the WMCHS, increases the PP required for the heat transfer coefficient and improves the temperature uniformity in the WMCHS.

## Figures and Tables

**Table 1:** Thermophysical properties of water, $Al_{2}O_{3}$ and CuO NP [40,40,42].

Properties	$H_{2}O$	$Al_{2}O_{3}$	**CuO**
$C_{P\;}\left(J/kg\cdot K\right)$	4179	765	540
k (W/m·K)	0.613	40	18
$\rho \;\left(kg/m^{3}\right)$	997.1	3970	6500
μ (kg/m·s)	0.001	-	-
$d_{S\;}\left(nm\right)$	-	13	100

**Table 2:** Pressure drop in heat sink at different Reynolds numbers for two different nanoparticles.

φ	Re = 500	Re = 1000	Re = 1500
Alumina			
0	74.78	231.2	466
1%	90.12	360.4	721.47
2%	110.25	389.31	835.52
Copper Oxide			
0	74.78	231.2	467
1%	91.15	368.45	733.24
2%	114.32	405.41	871.45
